# Note on the Treatment of Rupture of the Ligamentum Patellæ

**Published:** 1877-10-20

**Authors:** John Chiene

**Affiliations:** Assistant-Surgeon Edinburgh Royal Infirmary


### NOTE ON THE TREATMENT OF RUPTURE OF THE LIGAMENTUM PATELLÆ.

BY JOHN CHIENE, Esq., Assistant-Surgeon Edinburgh Royal Infirmary.

J. F., aged forty-four, was admitted to the Surgical Clinical Wards on the 8th of September, 1876. He limped into the hospital, complaining that something had given way in his right knee. He stated that, shortly before his admission, in going down a ladder his right foot caught on the last step, and that he fell forwards, his right leg bending under him. On examining the joint, the nature of the accident was at once evident. The patella lay on the anterior surface of the femur above the condyles; there was a distinct gap between the patella and the tubercle of the tibia. The ligamentum patellæ could not be felt. The patient could not extend his leg.

*Treatment.*—An oblong piece of strong extension plaster, large enough to cover the anterior and lateral aspects of the thigh, was heated, and fixed in position by means of a roller bandage. It was shaped so as to embrace the patella, and to its distal corners, on either side of the knee-joint, two pieces of strong india-rubber tubing, eight inches in length, were attached by tapes. The limb was then laid on a posterior splint with foot-piece, and slung in the inclined position to a wire cradle. The india-rubber tubing was fastened by means of tapes to the footpiece, and tightened sufficiently to bring the patella to its normal position. The elasticity of the india-rubber relieved the feeling of rigidity, and its contractility counteracted any loosening of the apparatus due to stretching of the tapes or slipping of the plaster.

The patient was in bed eight weeks. The plaster required renewal once. He never had any uneasiness from the apparatus, and was discharged on the 4th of November with firm union. Measurement from the upper border of the patella to the tubercule of the tibia was four inches on both limbs. On the 3d of January, 1877, the patient was shown at the Medico-Chirurgical Society, with complete restoration of flexion and extension at the knee-joint. The measurement from the upper border of the patella to the tubercle of the tibia was now 4½ inches, slight stretching of the newly formed material having taken place since 4th November.

I have also used this simple method in a case of fracture of the patella with an equally satisfactory result, simply drawing the upper fragment down to the lower, and applying nothing to fix or push up the lower fragment.—*Edinburgh Medical Journal*, February, 1877, p. 708.

